# 免疫检查点抑制剂相关糖尿病2例报道及文献回顾

**DOI:** 10.3779/j.issn.1009-3419.2021.102.54

**Published:** 2022-01-20

**Authors:** 尧尧 任, 琳琳 张, 瑜 王, 殿胜 钟

**Affiliations:** 1 300052 天津，天津医科大学总医院肿瘤科 Department of Oncology, Tianjin Medical University General Hospital, Tianjin 300052, China; 2 300052 天津，天津医科大学总医院内分泌科 Department of Endocrinology, Tianjin Medical University General Hospital, Tianjin 300052, China

**Keywords:** 肺肿瘤, 免疫检查点抑制剂, 糖尿病, Lung neoplasms, Immune checkpoint inhibitor, Diabetes mellitus

## Abstract

免疫检查点抑制剂在临床应用日益广泛，少见不良反应的发生日益增多。本文的目的是进一步提高对免疫检查点抑制剂相关糖尿病的认识。我们报道2例免疫检查点抑制剂相关糖尿病病例。对免疫检查点抑制剂相关糖尿病进行文献复习，讨论相关临床特点、可能的机制及最佳治疗方式。2例患者均为老年女性，1例在使用程序性死亡受体-配体1（programmed cell death-Ligand 1, PD-L1）单抗7个月后出现血糖增高，1例在使用PD-1单抗6周后出现糖尿病酮症酸中毒。2例患者诊断明确后均使用外源性胰岛素控制血糖。病例1持续使用免疫检查点抑制剂至今，病例2未再接受免疫治疗。人类白细胞抗原（human leukocyte antigen, HLA）基因型等因素可能解释了某些个体出现免疫检查点抑制剂相关糖尿病的危险原因。免疫检查点抑制剂相关糖尿病是一种并不少见，但可以危及生命的内分泌系统不良反应，需要医生提高警惕。使用免疫检查点抑制剂的患者需要进行血糖监测，如果出现血糖异常应及时请内分泌专科医生协助诊治。

## Abstract (en)

Immune checkpoint inhibitors (ICIs) are widely used in clinic, and the incidence of rare adverse events are increasing. The aim of this paper is to better define the rare adverse effect of diabetes mellitus associated with ICIs. We report 2 cases of diabetes mellitus associated with ICIs. Literature review was conducted and we discussed the clinical presentation, potential mechanisms and suggestions for optimal management. Two patients were both elderly women, case 1 had increased blood glucose after 7 months of using Durvalumab, and cases 2 had diabetic ketoacidosis after 6 weeks of using Pembrolizumab. Both patients were administered exogenous insulin to control blood glucose. Case 1 has been treated with Durvalumab until now and case 2 discontinued using of Pembrolizumab. HLA genotypes and other factors may explain the risk factors of diabetes associated with ICIs in some individuals. Diabetes mellitus associated with ICIs is an uncommon but potentially life-threatening endocrine system adverse event, which requires doctors to be vigilant. The patients who use ICIs need to monitor blood glucose. If they have hyperglycemia, endocrinologists should be asked to assist in diagnosis and treatment.

免疫检查点抑制剂（immune checkpoint inhibitors, ICIs）作为一类新型抗肿瘤药物，已经在恶性黑色素瘤、肺癌、尿路上皮癌等恶性肿瘤治疗中表现出显著疗效。自2011年上市以来，随着应用的普及，不良反应也逐渐被了解。由于ICIs特定的作用目标及机制，可引起自身免疫及炎症效应，也被称为免疫相关不良反应（immune-related adverse effects, irAEs）。与传统化疗的不良反应不同，irAEs表现隐匿且涉及身体各个系统，某些严重不良反应会导致患者中（终）止治疗，甚至威胁生命。

由于ICIs阻断T细胞上的抑制分子使T细胞活化，活化的T细胞除攻击肿瘤细胞外，会破坏免疫耐受从而与自身抗原发生反应。在涉及程序性死亡受体-1（programmed cell death-1, PD-1）/细胞毒T淋巴细胞相关抗原4（cytotoxic T lymphocyte-associated antigen-4, CTLA-4）单克隆抗体的临床试验中均有自身免疫性内分泌疾病的报道，多为垂体炎、甲状腺功能异常，对于自身免疫性糖尿病的报道较少。

## 1 病例资料

病例1，女性，71岁。2020年2月因“咳嗽伴消瘦1月”就诊。胸部计算机断层扫描（computed tomography, CT）示右肺门增大，纵隔多发增大淋巴结融合，考虑肿瘤性病变。腹部CT示右侧肾上腺结节，不除外转移。颈部CT示双侧锁骨上窝肿大淋巴结，以右侧为著。脑部磁共振成像（magnetic resonance imaging, MRI）示多发脑转移瘤。行B超引导下颈部淋巴结穿刺活检术，病理：小细胞癌，考虑转移性，Ki-67 index约90%。既往吸烟史40年，平均40支/日；否认糖尿病、自身免疫性疾病及家族遗传病史。入院诊断：广泛期小细胞肺癌。2020年3月开始给予依托泊苷+卡铂+度伐利尤单抗治疗。4个周期疗效评价为部分缓解（partial response, PR）（图 1）。行全脑放疗同时度伐利尤单抗维持治疗。2020年9月因肺部肿块较前略增大（图 2A），肾上腺转移增大（图 2B）给予局部放疗（肺部、肾上腺）并继续度伐利尤单抗维持治疗。2020年11月患者不明原因血糖增高（图 3），无恶心、呕吐、乏力、烦渴多尿、精神症状、视力下降、四肢麻凉等，无发热、感染等应激状况。院外自测空腹血糖15.4 mmol/L-30 mmol/L。入院查空腹血糖13.92 mmol/L；糖化血红蛋白9.8%；C肽0.14 ng/mL；胰岛细胞抗体（islet cell antibody, ICA）（-）、谷氨酸脱羧酶抗体（glutamic acid decarboxylaseantibody, GADA）（-）（表 1）。排除自发性1型糖尿病、2型糖尿病，考虑特发性糖尿病（药物相关）。诊断：ICI相关糖尿病，不良事件通用术语标准（common terminology criteria adverse events, CTCAE）2级。给予甘精胰岛素、谷赖胰岛素控制血糖。血糖平稳后，继续度伐利尤单抗维持治疗至今，且仍使用甘精胰岛素、谷赖胰岛素控制血糖，空腹血糖维持5.5 mmol/L-9.6 mmol/L。

**图 1:**
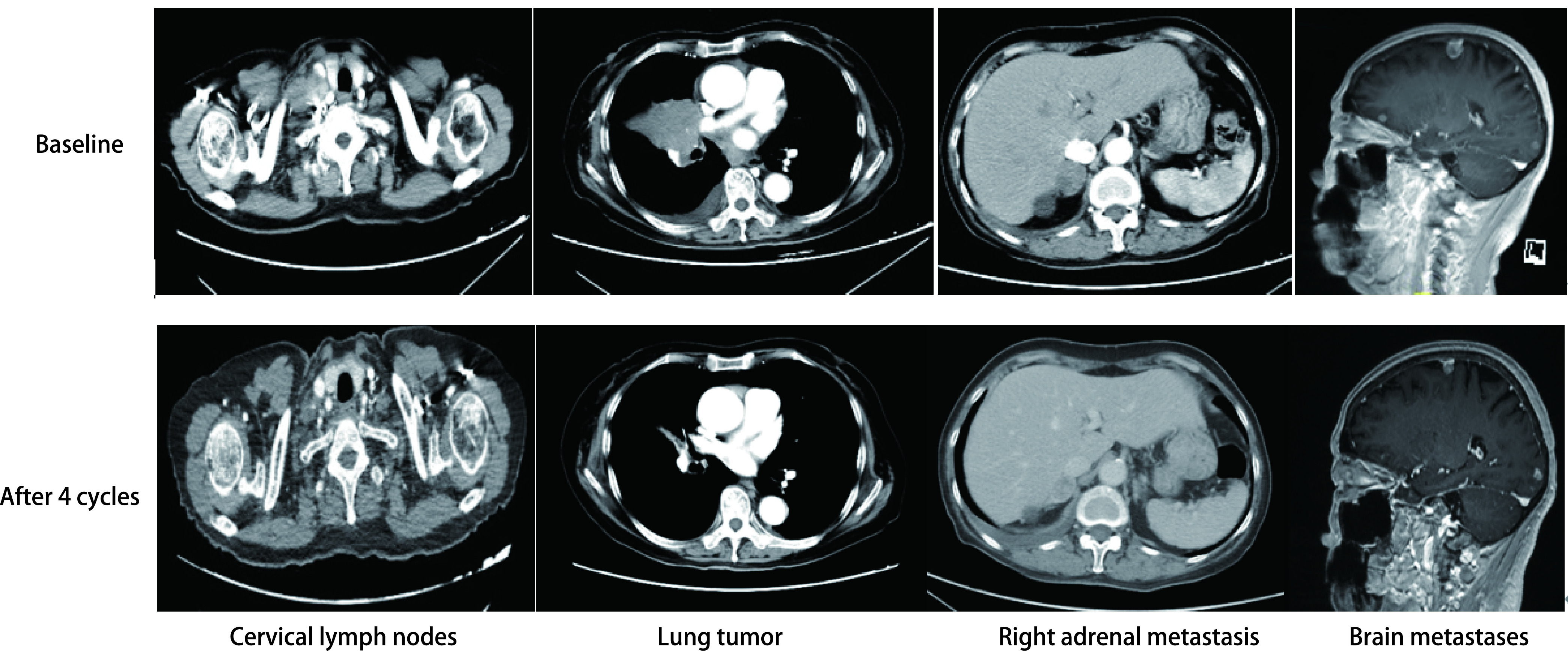
病例1基线影像及4个周期治疗后疗效评价 Baseline image of case 1 and efficacy evaluation after 4 cycles of treatment

**图 2:**
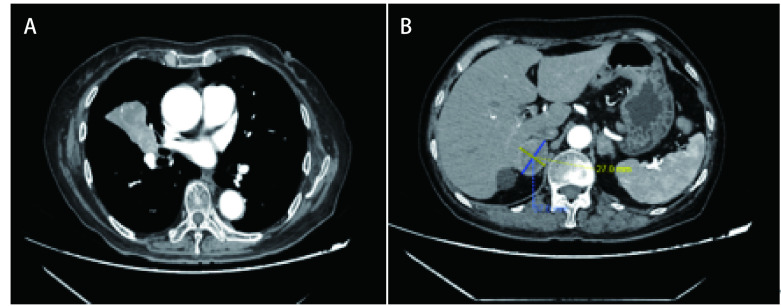
维持治疗期间出现肺部、肾上腺寡进展。A：胸部CT示肺门病灶较前增大；B：腹部CT示肾上腺转移较前增大。 Lung and adrenal metastases during maintenance treatment. A: chest CT showed hilar lesions larger than before; B: abdominal CT showed adrenal metastases larger than before. CT: computed tomography.

**图 3:**
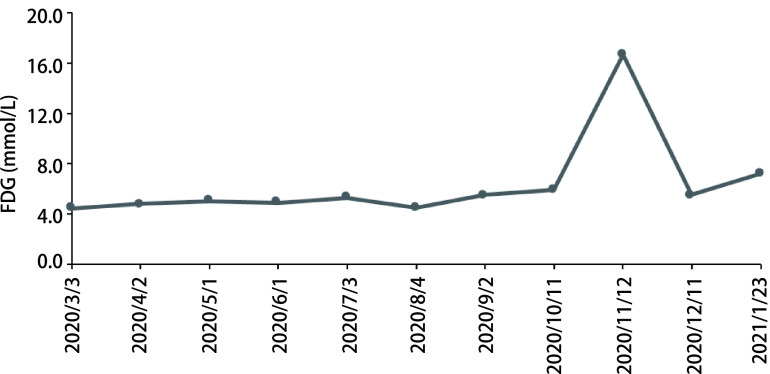
病例1空腹血糖变化曲线 Curve of fasting plasma glucose (FPG) in case 1

**表 1:** 患者化验检查表 Biochemical data of two cases

Index	Case 1					Case 2	Ref.
	Fasting	0.5 h	1 h	2 h	3 h		
Palsma glucose (mmol/L)	13.92	16.66	22.65	30.76	32.75	NE	3.6-5.8
Palsma insulin (mU/L)	12.5	11.9	11.6	13.6	12.7	NE	4-18
C-peptide (ng/mL)	0.14	0.13	0.24	0.45	0.44	< 0.01	0.78-5.19
HbA1c (%)	9.8					7.8	4-6
Urine protein	-					-	-
KET	-					4+	-
Arterial blood gas pH	NE					6.98	7.35-7.45
fT4 (pmol/L)	12.54					13.16	9.01-19.05
Cor (*μ*g/dL)	20.10					24.8	5-25
ACTH (pg/mL)	14.3					49.2	0-46
IAA	-					+-	
ICA	-					-	
GADA	-					-	
HLA-DR4	NE					+	
NE: not examined; Ref: reference; KET: ketone; HbA1c: glycated haemoglobin; HLA: human leukocyte antigen; fT4: free thyroxine; Cor: cortisol; ACTH: adrenocorticotropic hormone; IAA: insulin autoantibody; ICA: islet cell antibody; GADA: glutamic acid decarboxylaseantibody; HLA: human leukocyte antigen. Ref: reference.							

病例2，患者，女性，61岁。2018年因颈部淋巴结肿大就诊。胸部CT示右肺门偏上方软组织增厚，右上肺支气管狭窄伴周围肿物影，伴纵隔侵犯及右上肺阻塞性炎性改变；右肺门及纵隔内淋巴结肿大。颈部淋巴结穿刺活检显示低分化腺癌，免疫组化支持肺来源。驱动基因检测阴性。给予贝伐珠单抗+培美曲塞+卡铂治疗5周期，后培美曲塞+贝伐珠单抗维持。2019年12月右颈部淋巴结增大、肾上腺新发转移灶，疗效评价为疾病进展，结合患者既往程序性死亡配体1（programmed cell death-ligand 1, PD-L1）肿瘤细胞阳性比例评分（tumor proportion score, TPS）为60%，给予帕博利珠单抗200 mg+贝伐珠单抗400 mg治疗。2个周期治疗后右颈部包块明显缩小。2020年2月1日患者晚餐后出现呕吐，呕吐物为内容物，夜间出现呼吸困难，次日晨起症状加重，伴有意识不清，逐渐昏迷。急诊化验尿酮体4+，血气pH 6.98，血糖29.8 mmol/L。考虑糖尿病酮症酸中毒（diabetic ketoacidosis, DKA）予补液、胰岛素泵降糖、纠酮等治疗后症状缓解。行胰腺CT平扫未见明显异常；C肽 < 0.01 ng/mL；人类白细胞抗原（human leukocyte antigen, HLA）-DR4阳性。（表 1）。诊断为ICIs相关糖尿病，CTCAE 4级。给予德谷胰岛素及谷赖胰岛素治疗，皮下血糖仪监测血糖。血糖稳定后患者更换化疗方案，未再使用帕博利珠单抗。

## 2 讨论

### 2.1 流行病学

ICIs相关糖尿病发生率在随机临床研究中报道为0.2%^[1]^，真实世界约0.9%（27/2, 960）^[2]^。平均发病年龄61岁（22岁-84岁），亚裔约占15%。发病患者用药方案包括单药PD-1单抗（65/91, 71%）、PD-L1单抗（7/91, 8%）、CTLA-4单抗（3/91, 3%）、CTLA-4联合PD-1（14/91, 15%）。一般发生在用药后4.5个周期（1个-17个），联合用药出现时间早于单药治疗（2.7个周期，范围：1个-5个）^[3]^。

我们的病例均为亚裔女性，糖尿病确诊年龄 > 60岁，并且既往均无糖尿病史。病例1使用的度伐利尤单抗为PD-L1单抗，根据既往文献报道PD-L1单抗诱发糖尿病的发生率低于PD-1^[4]^。病例1由于没有糖尿病史及相关症状，没有常规监测随机及餐后血糖，只是在空腹血糖检查时偶然发现血糖增高，对于部分患者存在空腹血糖正常而餐后血糖异常的情况，并且该患者在确诊糖尿病时糖化血红蛋白已有升高，不排除患者糖尿病发病时间早于7个月的可能。病例2在使用PD-1单抗6周后出现ICI相关糖尿病，与文献报道一致。

### 2.2 诊断及鉴别诊断

ICIs相关糖尿病临床表现无特异性，个体差异显著，轻者仅表现为血糖升高，严重者可出现DKA。诊断依据为：若患者使用ICIs前血糖正常，治疗后满足以下三条之一时即可诊断：①典型糖尿病症状（高血糖所导致的烦渴、多饮、多尿、体重减轻）或皮肤瘙痒、视力模糊等急性代谢紊乱的临床表现并且随机血糖≥11.1 mmol/L；②空腹血糖≥7.0 mmol/L；③口服葡萄糖耐量试验（oral glucose tolerance test, OGTT）2 h血糖≥11.1 mmol/L^[5]^。我们报道的2例患者在使用免疫治疗前血糖均正常，用药后空腹血糖升高，符合诊断标准。

既往报道的ICIs相关糖尿病患者中至少有一种胰岛自身抗体阳性者，占53%（47/88），而两种或两种以上的自身抗体阳性者占15%（13/88）。GADA阳性率为51%，ICA阳性率为13%，IAA阳性率为26%，锌转运蛋白8阳性率为4%。GADA阳性患者的平均发病时间更短，为3.1个周期（范围：1个-17个）^[3]^。我们报道的病例仅病例2 IAA为弱阳性，其余抗体均为阴性。在ICIs相关糖尿病诊断标准中，抗体不作为确诊依据。

ICIs相关性糖尿病与自发性1型糖尿病存在一定差异（表 2）。我们报道的2例患者，药物暴露史明确，且确诊糖尿病年龄与自发性1型糖尿病患者差别较大。化验室检测C-肽水平均低于检测下限，并且始终没有恢复。在诊断糖尿病过程中2例患者腹部CT没有显示胰腺异常表现。病例2存在HLA-DR4阳性，可能是其出现爆发型糖尿病表现的原因。

**表 2:** ICIs相关性糖尿病与自发性1型糖尿病的鉴别 Differentiation between ICIs related diabetes mellitus and spontaneous type 1 diabetes mellitus

Index	ICIs related diabetes mellitus	Spontaneous T1DM
Median age	61 years (ascribed to the incidence of cancer)	Young age
ICI therapy	Yes	No
onset	Slow, latent or fulminant	Acute
C-peptide level	Extremely low or undetectable	Increased after exogenous stimulation
Diabetes-related autoantibodies	50%	90%
HLA genotypes	Lower than spontaneous T1DM	≥90%
Treatment	Insulin	Insulin
ICIs: immune checkpoint inhibitors; T1DM: type 1 diabetes mellitus; HLA: human leukocyte antigen.		

### 2.3 机制

ICIs相关糖尿病多见于接受抗PD-1或PD-L1治疗的患者，这种特定ICIs倾向于影响特定的内分泌器官的现象可能暗藏了irAEs的机制。例如PD-1/PD-L1单抗甲状腺功能障碍更常见，伊匹木单抗更易出现垂体炎。不同ICIs引起irAE发生率不同的原因尚不清楚，但可能与靶组织对损伤或炎症的反应或ICIs对自身反应性T细胞的影响有关^[9]^。ICIs相关糖尿病患者随机胰高血糖素水平没有降低，表明损伤的靶细胞为胰岛β细胞，而*α*细胞没有受到影响^[2]^。

动物实验发现非肥胖糖尿病（non-obese diabetes, NOD）小鼠在免疫性糖尿病的发病进程中，β细胞上PD-L1表达增加^[10]^。缺乏PD-1的NOD小鼠会迅速发展为自身免疫性糖尿病^[11]^。PD-L1在β细胞中表达，而PD-1受体在T细胞中表达，相互作用的PD-1/PD-L1抑制自身反应性T细胞的激活，从而保护自身避免免疫性糖尿病^[12]^。给NOD小鼠注射PD-1/PD-L1单抗可导致糖尿病的发生，并伴有特异性CD8^+^ T细胞介导的广泛破坏性胰岛炎^[13]^。Guleria等^[14]^研究表明，与野生型NOD对照组相比，PD-L1/PD-L2缺陷型NOD小鼠的胰腺中会积聚CD8^+^ T细胞，从而诱导针对胰岛β细胞的自身免疫反应并快速破坏胰岛β细胞。CTLA-4单抗治疗的患者较少诱发糖尿病的原因在于其配体为CD80和CD86^[15]^。

### 2.4 治疗及转归

依据《免疫检查点抑制剂引起的内分泌系统免疫相关不良反应专家共识》^[5]^，对于ICIs相关性糖尿病应尽早启动胰岛素治疗，并且需对患者进行饮食、生活方式及血糖监测等方面的宣教。糖尿病的发生不是继续PD-1或PD-L1单抗治疗的禁忌，患者可以在胰岛素治疗的同时继续ICIs治疗。病例1经过内分泌专家指导治疗，通过短效和长效胰岛素的使用控制了血糖，并继续度伐利尤单抗治疗，目前仍在治疗中。病例2在使用帕博利珠单抗2个周期后突然发生DKA，考虑为爆发型糖尿病，并且该患者存在糖尿病敏感基因*HLA-DR4*。由于患者irAE分级为4级，停止了帕博利珠单抗使用，但停药后依然需要外源性胰岛素控制血糖。

通过临床病例及文献回顾，提醒我们在临床实践中应用免疫检查点抑制剂（特别是PD-1/PD-L1单抗），应常规监测静脉血糖、糖化血红蛋白及C肽水平。HLA基因型有助于早期识别ICIs相关性糖尿病的高危人群。在确诊ICIs相关性糖尿病后应尽早启动胰岛素治疗。ICIs相关糖尿病通常是永久性的，因此ICIs治疗停止后也应继续糖尿病的治疗和随访。血糖控制平稳后ICIs再重启问题需要肿瘤专家、内分泌专家共同讨论。
